# Perceived Stress Mediates the Relationship of Body Image and Depressive Symptoms in Individuals With Obesity

**DOI:** 10.3389/fpsyt.2019.00852

**Published:** 2019-11-20

**Authors:** Katrin Ziser, Carina Finklenburg, Simone Claire Behrens, Katrin Elisabeth Giel, Sandra Becker, Eva-Maria Skoda, Martin Teufel, Isabelle Mack, Stephan Zipfel, Florian Junne

**Affiliations:** ^1^Department of Psychosomatic Medicine and Psychotherapy, University Hospital Tuebingen, Tuebingen, Germany; ^2^Department of Psychosomatic Medicine and Psychotherapy, LVR-University Hospital, University Duisburg-Essen, Essen, Germany

**Keywords:** obesity, body image, mediation, depressive symptoms, cross-sectional

## Abstract

Obesity is a world-wide increasing condition classified by a BMI ≥ 30 kg/m^2^ that is frequently accompanied by various somatic comorbidities as well as an increased risk for mental comorbidities. Studies show associations of obesity with symptoms of depression, lower quality of life, and higher (perceived) stress compared to the general population. Body image has also been shown to play an important role in eating and weight disorders. The present study therefore aims to contribute to the understanding of the relationship of body image, perceived stress, and symptoms of depression in a morbidly obese population. *N* = 579 individuals with obesity were included upon presentation at a university clinic. The hypothesized mediating role of perceived stress in the relationship of body image dimensions and symptoms of depression could be confirmed. The results underline the importance of identifying promising stress management techniques and addressing perceived stress e.g. through mindfulness based approaches in the (lifestyle and/or weight) interventions for obesity taking into account the specific stressors of obesity affected individuals such as body image.

## Introduction

The prevalence of obesity has constantly increased in past years (1). The World Health Organization categorizes the severity of obesity in three classes: Having a body mass index (BMI) between 30 and 34.9 kg/m^2^ corresponds to class I obesity, a BMI between 35 and 39.9 as class II obesity, and a BMI ≥40 as class III obesity (2). Obesity is often accompanied by various somatic comorbidities such as diabetes mellitus, hypertension or fat metabolism disorders (3). Additionally, there is an increased risk for mental comorbidities such as depression or eating disorders (1).

In case of the potential comorbidity of depression, the association between obesity and depressive symptoms seems to be of reciprocal nature (4). There is evidence that symptoms of depression predict the development or maintenance of obesity in the long term (5). On the other hand, there is evidence that obesity increases the risk for depression (4, 6). Some studies suggested a linear association between severity of obesity and increased risk of depression and decreased quality of life (7). Other studies, however, did not replicate this observation and suggested a more complex association (8). A possible explanation for these incoherent observations is that psychological variables rather than weight alone drive the associations between BMI and depression.

A psychological variable that is closely related to BMI is body image, i.e. the psychological representation of one’s body. Body image can be defined as a construct consisting of different components: perception, attitudes, affect, and cognitions about/toward the size, shape, and form of one’s own body (9–11). In early literature, it was assumed that obese people might be unaware of their excess body weight and therefore don’t take countermeasures to normalize it (12). The current view is that obese people are more frequently dissatisfied with their bodies compared to normal-weight people, this difference being significantly higher in women compared to men (13) and body dissatisfaction and drive for thinness affect perceived stress in individuals with obesity (14). Reasons for higher body dissatisfaction in obesity are speculative but may originate from sociocultural factors such as current body ideals, stigmatization, or teasing by others (13). Somatic comorbidities or consequences of high weight such as restricted mobility and therefore restricted opportunities for social participation may also be of importance. Weight loss has been shown to potentially improve body image (15). Again, there does not seem to be a linear association between body dissatisfaction and severity of obesity (16), suggesting a complex modulation that takes other associated variables into account e.g. as a mediator.

One of these variables may be stress. Stress is a concept consisting of two major components: environmental conditions serving as stressors and a person’s appraisal of stress (17). Since personal appraisal has been more in focus (18), we refer to perceived stress in this manuscript as opposed to e.g. a physiological stress reaction. Recent studies show that increased symptoms of depression and a lower quality of life are associated with higher (perceived) stress in individuals with obesity compared to the general population (7, 19). Associations between different types of stress (e.g. chronic stress) and higher food intake as well as subsequent weight gain could be shown: Higher stress was associated with higher food intake (20–22). A possible explanation for these findings can be increased cortisol levels due to physiological stress reactions that may lead to the selection of more calorie-dense foods and therefore increase food intake (23). Perceived stress can therefore be seen as a barrier for successful weight management in obesity, potentially contributing to further weight gain (14). It seems at hand that an association between perceived stress and body image is likely and has been shown for body dissatisfaction and perceived stress in a large sample of adolescent girls (24). To our knowledge, however, it is so far unknown how body image, perceived stress, and depressive symptoms might interact in the context of weight management.

The present study therefore aims to further investigate the relationships between stress (symptoms of), depression, and body image in a cross-sectional sample of obese individuals. Based on the above reported previous findings, obesity, and depression are related although there might not be a direct linear association. However, body image, a psychological construct closely related to BMI, has been shown to be related to obesity and perceived stress. Taken these findings together, we deem it possible that a negative/disturbed body image may be a driving factor potentially leading to the development of a comorbidity of depression in patients with obesity and that the risk for developing depressive symptoms might be dependent on the amount of stress these individuals experience. To the best of our knowledge, this has not been tested before. The investigation therefore had the following hypothesis: The relationship of body image with symptoms of depression is mediated by perceived stress in individuals with obesity.

## Materials and Methods

### Sample

Individuals presenting themselves at an interdisciplinary assessment and consultation platform for patients with obesity at a tertiary university clinic were invited to participate in the study over a period of four years. Inclusion criterion was BMI ≥ 30 kg/m^2^, no exclusion criterion applied other than a substantial language barrier.

### Measures

The following questionnaires in paper-and-pencil versions were presented to all participants.

#### Perceived Stress Questionnaire (PSQ-20)

The PSQ-20 is a measure to assess perceived stress during the last four weeks (17, 25). It consists of 20 items, each scoring on one of the four subscales “worries,” “tension,” “joy,” and “demands.” An overall score describing the overall perceived stress level can be computed. The PSQ overall score is trimmed to range between 0 and 1. Internal consistency of the overall score in the present sample proved to be good with Cronbach’s α = .84.

#### Body Image Questionnaire (BIQ-20)

The BIQ-20 is a measure to assess body image on the two subscales “negative evaluation of the body” (NEB) referring to one’s negative attitude toward the own body and “perception of body dynamics” (PBD) referring to ones perception of personal vitality (26). It comprises a total of 20 items and has been validated within healthy and clinical populations (27, 28). Internal consistencies in the present sample proved to be good with Cronbach’s α = .87 for the NEB scale and α = .82 for the PBD scale.

#### Patient Health Questionnaire—Section Depression (PHQ-9)

The PHQ-9 section of the Patient Health questionnaire assesses depressive symptoms and their severity with nine items (29). A sum score between 0 and 4 corresponds to no signs of symptoms of depression, 5 to 9 mild severity of symptoms of depression, 10 to 14 medium severity, 15 to 19 pronounced symptoms of depression and 20 to 27 severe symptoms of depression. Internal consistencies in the present sample proved to be good with Cronbach’s α = 88.

### Procedure

Individuals were invited to participate in the study consecutively upon presentation at the clinic. They were informed about the study and if they consented to participate, were given the questionnaires und subsequently weighed. Somatic comorbidities were retrieved from the consultation documentation. This study was carried out in accordance with the recommendations of good clinical practice. The protocol was approved by the ethics committee of the medical faculty of the University of Tuebingen (No. 727/2012BO2). All subjects gave written informed consent in accordance with the Declaration of Helsinki.

### Statistical Analyses

All statistical analyses were performed in IBM SPSS Statistics (version 25). The level of significance for all analyses was set at α = .05. Means, standard deviations, and percentages are reported for sample descriptions. Since most of the variables are not normally distributed, Spearman rho correlations are reported for the associations between variables. The mediation analysis was performed according to the approach by Hayes (30) utilizing the PROCESS macro for SPSS (version 3.1). Direct and indirect effects are quantified using ordinary least square (OLS) regression-based path analysis. Inference about the indirect effect is determined by bootstrapping, reporting 95% bootstrap confidence intervals [95% BCa CI]. The number of bootstrap samples was set at 10,000. Effects of the mediation analysis are reported as unstandardized effects (*b*).

## Results

A total of *N* = 663 individuals agreed to participate in the study. After omitting data sets with missing values for one of the investigated measures, *n* = 579 participants were included in the final analyses.

### Descriptive Statistics

For an overview of the descriptive statistics, see Table 1. Notably, with a mean at the top of the mild severity range, our study sample shows more depressive symptoms compared to the German population norms (*M* = 3.56, *SD* = 4.08, 31). This applies to the PSQ-20 overall score and the BIQ subscales as well. The mean scores of NEB and PBD ranged around the 99^th^ and 14^th^-21^st^ percentile respectively, compared to representative German population norms (27). The PSQ-20 overall score was also higher than the reference value of *M* = 0.33 of healthy adults (17).

**Table 1:** Demographic characteristics of the study population (N = 579).

Variable	M (SD)	%
Gender		70.3 female
		20.9 male
Age (years)	41.68 (12.21)	
BMI (kg/m^2^)	45.52 (8.19)	
Obesity - Class I - Class II - Class III		7.1 18.3 74.6
Comorbidities - Type 2 diabetes - Arterial hypertension - Hyperlipidemia - Hyperuricemia - Hypothyroidism		31.4 54.9 30.8 12.0 24.3
PHQ-9/symptoms of depression	9.43 (6.12)	
BIQ-20 - NEB - PBD	37.48 (8.54) 26.09 (6.86)	
PSQ-20 - Overall score - Worries - Tension - Joy - Demands	0.48 (0.20) 0.44 (0.25) 0.49 (0.24) 0.45 (0.24) 0.42 (0.22)	

BIQ-20, Body Image Questionnaire; BMI, body mass index (kg/m^2^); PHQ-9, Patient Heath Questionnaire-9 (section depression); PSQ-20, Perceived Stress Questionnaire.

Most of the study population classified as class III obesity. This probably results from the clinical setting participants were recruited: Presenting oneself at a university hospital to seek consultation and/or treatment for obesity often implies psychological strain because of the weight status. Psychological strain is more likely for severely obese individuals who experience more comorbidities and possible impairments (Table 1).

### Correlations

For the complete correlation matrix, see Table 2. Correlations were found in the expected directions: Perceived stress and depressive symptoms do not correlate with BMI whereas there are significant but small correlations for BMI and both body image dimensions (Table 2).

**Table 2:** Spearman rho (*r*_s_) correlations of investigated variables.

Variable	Stress	NEB	PBD	BMI
Depressive symptoms (PHQ-9)	.722**	.456**	−.460**	−.015
Stress (PSQ-20)	—	.477**	−.355**	−.064
NEB (BIQ-20)	—	—	−.356**	.175**
PBD (BIQ-20)	—	—	—	−.169**

**p < .01; BIQ-20, Body Image Questionnaire; BMI, body mass index (kg/m^2^); NEB, negative evaluation of the body; PBD, positive body dynamics; PHQ-9, Patient Health Questionnaire (section depression); PSQ-20, Perceived Stress Questionnaire.

There is also a significant correlation between perceived stress and depressive symptoms. Both constructs are a sort of psychological distress and therefore associated but not the same construct. Stress may increase the risk for developing depressive symptoms.

### Mediation Model

**Figure 1:**
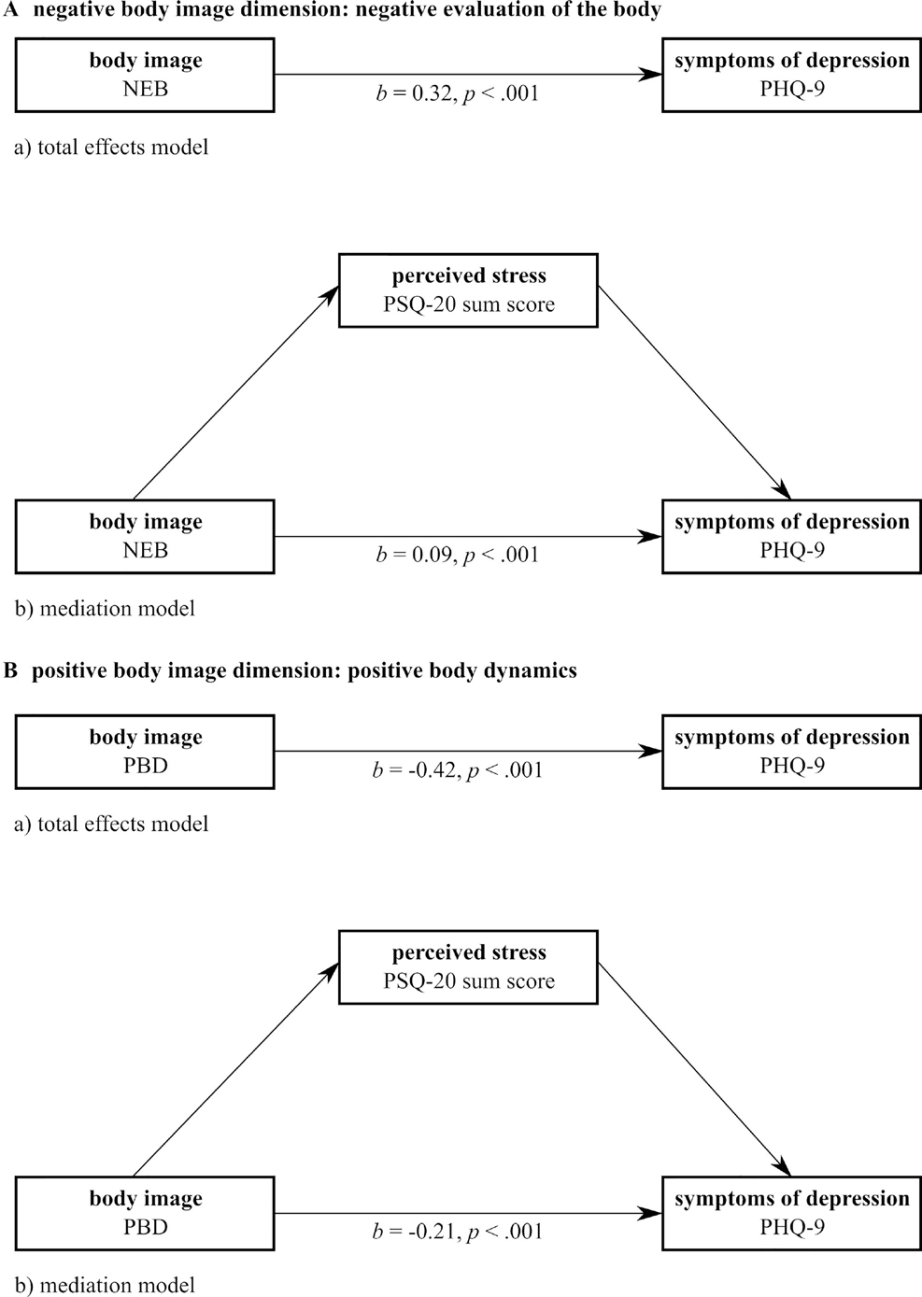
Graphical illustration of the mediation analyses. NEB, negative evaluation of the body; PBD, positive body dynamics; PHQ-9, Patient Health Questionnaire (section depression); PSQ-20, Perceived Stress Questionnaire.

The total effects model for NEB and symptoms of depression proved to be significant [*F*(1,577) = 138.42, *p* < .001, *R* ^2 ^= 0.19]. In line with the found correlations, NEB and symptoms of depression are positively associated, indicating that a more NEB is associated with higher symptoms of depression. This relationship explained about 19% of variance in symptoms of depression. The mediation model with perceived stress as the mediator between NEB and symptoms of depression also proved to be significant [*F*(2,576) = 304.52, *p* < .001, *R* ^2 ^= 0.51] indicating a significant mediation. The direct effect of NEB on symptoms of depressions is still a significant pathway, although to a smaller degree (total effects model: *b* = 0.32, mediation model: *b* = 0.09). The indirect effect [*b* = 0.23, (95% BCa CI) (0.19; 0.27)] depicts the influence of NEB on symptoms of depression mediated by perceived stress and proved to be significant. The mediation model explained about 51% of variance (**Figure 1**).

The mediation analysis for PBD showed similar results. The total effects model proved to be significant [*F*(1,577) = 159.38, *p* < .001, *R* ^2 ^= 0.22] indicating a negative association between the two variables. Hence, a higher perception of one’s vitality is associated with less symptoms of depression. The mediation model with perceived stress as a mediator between PBD and symptoms of depression was also significant [*F*(2,576) = 349.61, *p* < .001, *R* ^2 ^= 0.55]. Similar to the first mediation model, the direct effect of PBD on symptoms of depression remained significant but to a smaller degree (total effects model: *b* = −0.42, mediation model: *b* = −0.21). The indirect effect proved significant as well [*b* = −0.21, 95% BCa CI (−0.26; −0.16)]. This mediation model accounted for 55% of variance.

In consideration of potential gender differences in our analysis, we computed the examined correlations and mediation analyses separately for the male and female subgroups. The only difference in correlations was a non-significant correlation for BMI and PBD in the male subgroup. All other correlations corresponded to the correlations of the whole group. The mediation analyses for the male and female subgroup both corresponded to the mediation analysis for the whole group.

## Discussion

To the best of our knowledge, this is the first study to explore the mediating role of perceived stress in the relationship of body image dimensions and symptoms of depression in individuals with obesity. We found perceived stress to be a significant mediator for negative dimensions of body image (i.e. negative evaluation of the body) as well as positive body image dimensions (i.e. perception of body dynamics). The mediation model explained a good portion of variance of the relationships of body image dimensions, perceived stress and symptoms of depression in our obese study population.

In line with a review by Schwartz and Brownell (12), we found a linear association of BMI with the perception of body image dimensions in our study sample. Schwartz and Brownell concluded in their review that this association applies to the whole population of obese individuals and may not apply in subgroups such as obese women seeking weight loss. According to the authors, there was not sufficient evidence to comment on men. Looking only at the male subgroup in our study, linear associations between BMI and body image were significant for the negative dimension of body image (NEB) but not for the positive dimension of body dynamics. We can therefore expand existing research concerning this regard.

With regard to the confirmed mediation model, we found similar results before in individuals with anorexia nervosa for the NEB in a longitudinal design (32). In this study, because patients underwent treatment at the time of the perceived stress assessment, increased stress could have been a result of gaining weight during treatment. However, this explanation does not seem sufficient since we found similar patterns in obesity. There are similar patterns concerning other aspects of body image between individuals with obesity and with anorexia nervosa as well: Recent studies examining body image in individuals with obesity found evidence that obese individuals are not generally biased in estimating their own body size but cognitive-affective factors are likely to play an important role (33). Obese children and adolescents did also not differ in their body size estimation from normal weight children and adolescents (34). To the best of our knowledge, possible common mechanisms have still to be determined.

Concerning the treatment of obesity related body image disturbances, studies predominantly show an effect of weight loss and/or lifestyle interventions for the improvement of body image and symptoms of depression (15, 35, 36). Since long-term weight management in obesity remains difficult (37), evidence for identifying relevant variables and developing more specialized treatments is essential. The results of our study suggest that two important aspects should be addressed in order to help prevent the development of depressive symptoms: body image at first instance and also perceived stress.

Only few studies so far directly addressed body image in obese individuals in their intervention: Cognitive behavioral body image therapy lead to an improved body image (38), but did not lead to more improvement in body image when it was added to a weight control program (39). A pilot study showed an increase in body satisfaction in obese adolescents following a mirror exposure intervention (40). Given current research on mirror or body exposure interventions in the field of eating and weight disorders, it might be a recommended intervention for individuals with body image disturbances with and without an eating disorder (41).

In case of perceived stress, successful and specific (body image directed) stress management techniques for obese individuals are missing (22). From our point of view, mindfulness based strategies seem promising. They encourage a mindfulness, non-judgmental stance toward one’s body, include body related exercises (e.g. body scan) and reduce perceived stress (see for a systematic review).

A further consideration for this study would have been subgroup analysis for individuals with a binge eating disorder as it has been indicated before that this population shows especially pronounced body image disturbances (43, 44). In our sample, about 25% of participants screened positive for binge eating (episodes). However, as it was only a written screening and no structured and standardized assessed diagnosis, we deemed it not valid to make a subgroup analysis but would plan for a standardized clinical assessment in future studies.

Other studies indeed showed associations between binge eating, perceived stress, and symptoms of depression (12) and determined that binge eating patients are a distinct subgroup of the obese population considering these variables. In this study, we could show that these associations might also apply to the whole obese population, at least the severely obese (class III obesity). One might hypothesize that body image is an important aspect in obesity as well as binge eating. Perceived stress could therefore be an important factor deciding if or to what extent e.g. body image dissatisfaction has a negative impact on symptoms of depression in both entities.

There are three specific limitations to our study we want to address. First, it is a cross-sectional study. We therefore cannot deduct causal relationships between the investigated variables. However, considering the big sample size and robust effects, longitudinal validation of the presented associations (e.g. 32 for patients with anorexia nervosa), would be the next step.

Second, the investigated study population may not be representative for all patients with obesity. Due to the recruitment at a specialized unit for obesity at a university clinic, patients were at least seeking consultation (if not treatment) for obesity. Thus, our study population was more likely to be discontent with their weight and wanting to change it (either through bariatric surgery or conservative programs). The mean BMI of our study population reflects this as well: nearly 75% classified as class III obesity. However, somatic causes of obesity (such as hypercortisolism) could not be included as exclusion criterion in the present study, as well as chronic psychotic (e.g. mild delusional symptoms) or personality disorders. This could potentially bias the results. Considering the comparatively low prevalence of these causes and symptoms and our large sample size, such a bias does not seem likely, but could be controlled for in future studies.

Third, we used a measure for body image that operationalizes body image in a broad way on a two-dimensional score. One subscale represents a negative dimension of body image similar to body dissatisfaction and one dimension represents a positive dimension of body image such as body satisfaction or satisfaction with body appearance. Since this was the first study exploring the mediating role of perceived stress, we think this broader defined measure of body image was adequate but would encourage further research to assess more specific aspects of body image. It can help to understand which aspects of body image have the greatest impact on the here examined relations.

Further directions of research on this topic could include the whole weight spectrum from underweight to obesity determining if these findings can be replicated. If these findings cannot be replicated, there might be e.g. a U-shaped association between body image, perceived stress and/or symptoms of depression if the whole weight spectrum is taken into account as shown by Martin-Rodriguez et al. (8) for the association of BMI at baseline and new-onset of depression. In this case, the mediation model confirmed in this study would not be applicable in the normal weight range. This would point at (not-normal-ranged) weight as an important part of the underlying mechanisms of the found results. Subgroup analyses according to a manifested eating disorder would also be beneficial. Additionally, identifying the most promising stress management techniques, incorporating them or e.g. mindfulness based approaches into lifestyle interventions for obesity and verifying its efficacy for the specific target group of individuals with obesity remains an important challenge.

## Data Availability Statement

The datasets generated for this study are available on request to the corresponding author.

## Ethics Statement

The studies involving human participants were reviewed and approved by the ethics committee of the medical faculty of the University of Tuebingen. The patients/participants provided their written informed consent to participate in this study.

## Author Contributions

KZ, CF, SCB, KEG, MT, SZ, and FJ contributed to the conception and design of the study. SB, E-MS, and IM substantially contributed to the acquisition of data for the study. KZ performed the statistical analysis and wrote the first draft of the manuscript. All authors contributed to manuscript revision, read, and approved the submitted version.

## Funding

KZ is supported by the “Konrad Adenauer Stiftung” (Konrad Adenauer Foundation). The authors acknowledge support by the “Deutsche Forschungsgemeinschaft (DGF)” and the Open Access Publishing Fund of the University of Tuebingen.

## Conflict of Interest

The authors declare that the research was conducted in the absence of any commercial or financial relationships that could be construed as a potential conflict of interest.

## References

[1] Williams EP Mesidor M Winters K Dubbert PM Wyatt S Overweight and obesity: prevalence, consequences, and causes of a growing public health problem. Curr Obesity Rep (2015) 4(3):363–70. 10.1007/s13679-015-0169-4 26627494

[2] World Health Organization. Obesity: preventing and managing the global epidemic. Geneva, Switzerland: World Health Organization (2000). 11234459

[3] Guh DP Zhang W Bansback N Amarsi Z Birmingham CL Anis AH The incidence of co-morbidities related to obesity and overweight: a systematic review and meta-analysis. BMC Public Health (2009) 9(1):88. 10.1186/1471-2458-9-88 19320986 PMC2667420

[4] Luppino FS de Wit LM Bouvy PF Stijnen T Cuijpers P Penninx BW Overweight, obesity, and depression: a systematic review and meta-analysis of longitudinal studies. Arch Gen Psychiatry (2010) 67(3):220–9. 10.1001/archgenpsychiatry.2010.2 20194822

[5] Markowitz S Friedman MA Arent SM Understanding the relation between obesity and depression: causal mechanisms and implications for treatment. Clin Psychol: Sci Pract (2008) 15(1):1–20. 10.1111/j.1468-2850.2008.00106.x

[6] Roberts RE Deleger S Strawbridge WJ Kaplan GA Prospective association between obesity and depression: evidence from the Alameda County Study.Int J Obesity (2003) 27(4):514–21. 10.1038/sj.ijo.0802204 12664085

[7] Preiss K Brennan L Clarke D A systematic review of variables associated with the relationship between obesity and depression. Obesity Rev (2013) 14(11):906–18. 10.1111/obr.12052 23809142

[8] Martin-Rodriguez E Guillen-Grima F Aubá E Marti A Brugos-Larumbe A Relationship between body mass index and depression in women: a 7-year prospective cohort study. APNA study Eur Psychiatry (2016) 32:55–60. 10.1016/j.eurpsy.2015.11.003 26803616

[9] Slade PD Body image in anorexia nervosa. Br J Psychiatry (1988) 153:20–2. 10.1192/S0007125000298930 3072049

[10] Slade PD What is body image? Behav Res Ther (1994) 32(5):497–502. 10.1016/0005-7967(94)90136-8 8042960

[11] Farrell C Shafran R Lee M Empirically evaluated treatments for body image disturbance: a review. Eur Eating Disord Rev (2006) 14(5):289–300. 10.1002/erv.693

[12] Schwartz MB Brownell KD Obesity and body image. Body Image (2004) 1:43–56. 10.1016/S1740-1445(03)00007-X 18089140

[13] Weinberger N-A Kersting A Riedel-Heller SG Luck-Sikorski C Body dissatisfaction in individuals with obesity compared to normal-weight individuals: a systematic review and meta-analysis. Obesity Facts (2016) 9(6):424–41. 10.1159/000454837 PMC5644896 28013298

[14] Junne F Ziser K Giel KE Schag K Skoda E Mack I Determinants of perceived stress in individuals with obesity: exploring the relationship of potentially obesity-related factors and perceived stress. Obesity Facts (2017) 10(2):127–38. 10.1159/000454833 PMC5644933 28433993

[15] Chao H-L Body image change in obese and overweight persons enrolled in weight loss intervention programs: a systematic review and meta-analysis.PloS One (2015) 10(5):e0124036. 10.1371/journal.pone.0124036 25946138 PMC4422747

[16] Sarwer DB Thompson JK Cash TF Body image and obesity in adulthood. Psychiatr Clinics North America (2005) 28(1):69–87. 10.1016/j.psc.2004.09.002 15733612

[17] Fliege H Rose M Arck P Walter OB Kocalevent R-D Weber C The Perceived Stress Questionnaire (PSQ) reconsidered: Validation and reference values from different clinical and healthy adult samples. Psychosomatic Med (2005) 67(1):78–88. 10.1097/01.psy.0000151491.80178.78 15673628

[18] DeLongis A Coyne JC Dakof G Folkman S Lazarus RS Relationship of daily hassles, uplifts, and major life events to health status. Health Psychol (1982) 1(2):119–36. 10.1037/0278-6133.1.2.119

[19] Sarwer DB Lavery M Spitzer JC A review of the relationships between extreme obesity, quality of life, and sexual function. Obesity Surg (2012) 22(4):668–76. 10.1007/s11695-012-0588-1 22293982

[20] Torres SJ Nowson CA Relationship between stress, eating behavior, and obesity. Nutrition (2007) 23(11–12):887–94. 10.1016/j.nut.2007.08.008 17869482

[21] Dallman MF Stress-induced obesity and the emotional nervous system. Trends In Endocrinol Metab (2010) 21(3):159–65. 10.1016/j.tem.2009.10.004 PMC2831158 19926299

[22] Moore CJ Cunningham SA Social position, psychological stress, and obesity: a systematic review. J Acad Nutr Dietetics (2012) 112(4):518–26. 10.1016/j.jand.2011.12.001 22709702

[23] Adam TC Epel ES Stress, eating and the reward system. Physiol Behav (2007) 91(4):449–58. 10.1016/j.physbeh.2007.04.011 17543357

[24] Johnson F Wardle J Dietary restraint, body dissatisfaction, and psychological distress: A prospective analysis. J Abnormal Psychol (2005) 114(1):119–25. 10.1037/0021-843X.114.1.119 15709818

[25] Levenstein S Prantera V Varvo V Scribano ML Berto E Luzi C Development of the Perceived Stress Questionnaire: a new tool for psychosomatic research. J Psychosomatic Res (1993) 37(1):19–32. 10.1016/0022-3999(93)90120-5 8421257

[26] Clement U Löwe B Die Validierung des FKB-20 als Instrument zur Erfassung von Körperbildstörungen bei psychosomatischen Patienten.Psychother Psychosomatik Medizinische Psychol (1996) 46:254–9. 8765897

[27] Albani C Blaser G Geyer M Daig I Schmutzer G Bailer H Überprüfung und Normierung des “Fragebogen zum Körperbild” (FKB-20) von Clement und Löwe (1996) an einer repräsentativen deutschen Bevölkerungsstichprobe. Z für Medizinische Psychol (2006) 15(3):99–109.

[28] Lamadé W Friedrich C Ulmer C Basar T Wei ß H Thon K-P Impact of body image on patients’ attitude towards conventional, minimal invasice, and natural orifice surgery. Langenbeck’s Arcjoves Surg (2011) 396(3):331–6. 10.1007/s00423-010-0669-3 20602112

[29] Kroenke K Spitzer RL The PHQ-9: a new depression diagnostic and severity measure. Psychiatr Ann (2002) 32(9):509–15. 10.3928/0048-5713-20020901-06

[30] Hayes AF Introduction to mediation, moderation and conditional process analysis: a regression-based approach. New York: The Guilford Press (2018).

[31] Rief W Nanke A Klaiberg A Braehler E Base rates for panic and depression according to the Brief Patient Health Questionnaire: a population-based study. J Affect Disord (2004) 82(2):271–6. 10.1016/j.jad.2003.11.006 15488257

[32] Junne F Wild B Resmark G Giel KE Teufel M Martus P The importance of body image disturbances for the outcome of outpatient psychotherapy in patients with anorexia nervosa: results of the ANTOP-study. Eur Eating Disord Rev (2019) 27(1):49–58. 10.1002/erv.2623 30009554

[33] Thaler A Geuss MN Mölbert SC Giel KE Streuber S Romero J Body size estimation of self and others in females varying in BMI. PloS One (2018) 13(2):e0192152. 10.1371/journal.pone.0192152 29425218 PMC5806871

[34] Mölbert SC Sauer H Dammann D Zipfel S Teufel M Junne F Multimodal body representation of obese children and adolescents before and after weight-loss treatment in comparison to normal-weight children. PloS One (2016) 11(11):e0166826. 10.1371/journal.pone.0166826 27875563 PMC5119783

[35] Dalle Grave R Cuzzolaro M Calugi S Tomasi F Temperilli F Marchesini G The effect of obesity management on body image in patients seeking treatment at medical centers. Obesity (2007) 15(9):2320–7. 10.1038/oby.2007.275 17890501

[36] Lasikiewicz N Myrissa K Hoyland A Lawton C Psychological benefits of weight loss following behavioural and/or dietary weight loss interventions. A Syst Res Rev Appetite (2014) 72:123–37. 10.1016/j.appet.2013.09.017 24075862

[37] Montesi L El Ghoch M Brodosi L Calugi S Marchesini G Dalle Grave R Long-term weight loss maintenance for obesity: a multidisciplinary approach. Diabetes Metab Syndrome Obesity: Targets Ther (2016) 9:37. 10.2147/DMSO.S89836 PMC4777230 27013897

[38] Rosen JC Orosan P Reiter J Cognitive behavior therapy for negative body image in obese women. Behav Ther (1995) 26(1):25–42. 10.1016/S0005-7894(05)80081-4

[39] Ramirez EM Rosen JC A comparison of weight control and weight control plus body image therapy for obese men and women. J Consult Clin Psychol: Sci Pract (2001) 69(3):440–6. 10.1037//0022-006X.69.3.440 11495173

[40] Jansen A Bollen D Tuschen-Caffier B Roefs A Tanghe A Braet C Mirror exposure reduces body dissatisfaction and anxiety in obese adolescents: A pilot study. Appetite (2008) 51(1):214–7. 10.1016/j.appet.2008.01.011 18342397

[41] Griffen TC Naumann E Hildebrandt T Mirror exposure therapy for body image disturbances and eating disorders: a review. Clin Psychol Rev (2018) 65(163-174). 10.1016/j.cpr.2018.08.006 30223161

[42] O’Reilly GA Coo kL Spruijt-Metz D Black DS Mindfulness-based interventions for obesity-related eating behaviours: a literature review. Obesity Rev (2014) 15(6):453–61. 10.1111/obr.12156 PMC4046117 24636206

[43] Lewer M Nasrawi N Schroeder D Vocks S Body image disturbance in binge eating disorder: a comparison of obese patients with and without binge eating disorder regarding the cognitive, behavioral and perceptual component of body image. Eating Weight Disord (2016) 21(1):115–25. 10.1007/s40519-015-0200-5 26178486

[44] Grilo CM Ivezaj V Lydecker JA White MA Toward an understanding of the distinctiveness of body image constructs in persons categorized with overweight/obesity, bulimia nervosa, and binge-eating disorder. J Psychosomatic Res (2019) 126:109757. 10.1016/j.jpsychores.2019.109757 PMC6842703 31522010

